# Roles of EP Receptors in the Regulation of Fluid Balance and Blood Pressure

**DOI:** 10.3389/fendo.2022.875425

**Published:** 2022-06-23

**Authors:** Lu Wang, Yiqian Wu, Zhanjun Jia, Jing Yu, Songming Huang

**Affiliations:** ^1^ Jiangsu Key Laboratory of Pediatrics, Children’s Hospital of Nanjing Medical University, Nanjing, China; ^2^ Nanjing Key Laboratory of Pediatrics, Children’s Hospital of Nanjing Medical University, Nanjing, China; ^3^ Department of Hematology and Oncology, Children’s Hospital of Nanjing Medical University, Nanjing, China; ^4^ Department of Nephrology, Children’s Hospital of Nanjing Medical University, Nanjing, China

**Keywords:** blood pressure, fluid metabolism, EP receptors, hypertension, kidney, prostaglandin E2, blood pressure (BP)

## Abstract

Prostaglandin E2 (PGE2) is an important prostanoid expressing throughout the kidney and cardiovascular system. Despite the diverse effects on fluid metabolism and blood pressure, PGE2 is implicated in sustaining volume and hemodynamics homeostasis. PGE2 works through four distinct E-prostanoid (EP) receptors which are G protein-coupled receptors. To date, pharmacological specific antagonists and agonists of all four subtypes of EP receptors and genetic targeting knockout mice for each subtype have helped in uncoupling the diverse functions of PGE2 and discriminating the respective characteristics of each receptor. In this review, we summarized the functions of individual EP receptor subtypes in the renal and blood vessels and the molecular mechanism of PGE2-induced fluid metabolism and blood pressure homeostasis.

## Introduction

Fluid metabolism is an important process that requires the expression of a number of hormones and mediators that act primarily through neuroendocrine mechanisms, ultimately affecting blood pressure homeostasis (1). Brain, cardiovascular system and kidney are remarkably precise and work in accordance in the control of water homeostasis and hemodynamic responses (2). In human, disorder of water homeostasis is associated with idiopathic and/or pathological alterations in physiological control, because many diseases induce defects in the complex mechanisms that control the intake and output of water and solute. The kidney, as one of the main organs controlling water balance and blood pressure, plays a major role in the ion and water metabolism.

**Figure 1:**
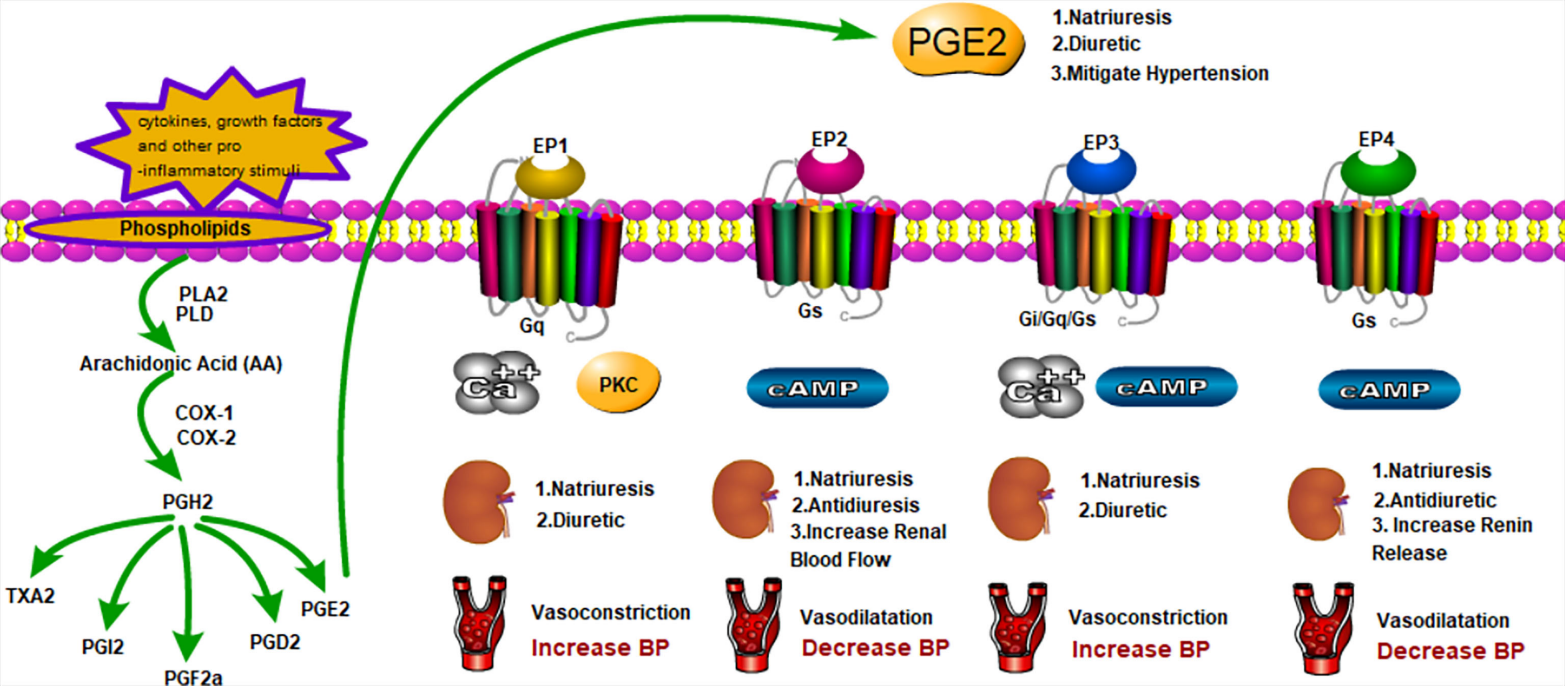
Generation of PGE2 and Functions in Fluid Balance and Blood Pressure with The EPs.

As a hormone-like chemical messenger derived from arachidonic acid (AA), prostaglandin (PGs)belong to a subclass of eicosanoids and are involved in a wide variety of biological functions in human body. The biosynthesis of prostaglandins was shown by **Figure 1**. AA is a polyunsaturated fatty acid that is present in esterified form in membrane phospholipids. In response to cytokines, growth factors and other pro-inflammatory stimuli, AA can be released by hydrolysis *via* the phospholipase A2, phospholipase D or phospholipase C pathways (3). They are then converted to PGs and leukotrienes (LTs) *via* the cyclooxygenase (COX) and lipoxygenase (LOX) pathways, respectively. COX catalyzes an initial cyclooxygenase reaction leading to the generation ofPGG2, followed by an endoperoxidase reaction reducing PGG2 to PGH2. The generated PGH2 can be converted to PGE2, PGD2, PGI2, PGF2α, and thromboxane A2 (TXA2) by individual prostanoid synthases (4). Since prostanoids are rapidly metabolically degraded, upon synthesis, they are released outside the cell and interact with specific G-protein-coupled receptors (GPCRs) in an autocrine or paracrine fashion. Besides the “classical” PGs, PGE2 and PGD2 can convert into bioactive cyclopentenone PG metabolites, PGA and PGJ2 respectively (5, 6). Kidney and vasculature are important targets of prostanoid action.

Thanks to their tremendous efficacy in treating pain, inflammation and fever through block the activity of both COX1 and COX2, non-steroidal anti-inflammatory drugs (NSAIDs) are the most frequently used drugs in the world today, accounting for nearly 5% of all prescription drugs (7). However, long-term use of NSAIDs may increase the risk of the gastric mucosal and small bowel injuries, cardiovascular disease, renal injury, respiratory tract inflammation and infection (8). All these deleterious effects on various organs can be explained by the inhibition of COX-dependent prostaglandin synthesis, which underlines the importance of COX and prostaglandins for systemic blood pressure and blood volume control (9–12). Among the different types of PGs, Prostacyclin (PGI2) is the main product of AA in vascular tissues. PGI2 is a vasodilator and inhibitor of platelet aggregation. However, the effects of PGI2 on dilating pulmonary vessels are greater than that on systemic circulation, PGI2 analogs have been used effectively in primary pulmonary hypertension (13). Whereas another arachidonic acid metabolite, Thromboxane A2(TXA2) promotes vasoconstriction and platelet aggregation. The balance between PGI2 and TXA2 in vascular homeostasis, and their imbalance in pregnancy-associated and neonatal vascular disorders has been well described (14). Prostaglandin E2 (PGE2)is the most abundant prostanoid detected in the kidney and a major prostanoid synthesized in vasculature, secondary to PGI2, and it exerts complex and diverse functions in maintaining fluid and blood pressure homeostasis (15). Revealing the role of PGE2 can provide important insight into paths for developing new therapies for treatment of body fluid imbalance and abnormal blood pressure. Contrarily, PGD2 is rarely synthesized in the kidney and aorta. There is little study on PGD2 in kidney and vasculature (15). Despite several studies have reported PGF2α plays a role in urine concentration and dilution, accumulating evidence indicates that the increase of PGF2α can be enzymatic conversion of PGE2 (16, 17). Therefore, PGE2 is considered to be the most important for the normal physiological function of maintaining fluid and blood pressure homeostasis.

In this review, we provide a summary of recent advances in PGE2 receptor research, by focusing on the molecular mechanism of PGE2-regulated fluid metabolism and blood pressure homeostasis.

## PGE2 and Fluid Balance

PGE2 is the main product of cyclooxygenase activity in all sections of the nephron, but the highest production is seen in the glomeruli and collecting ducts and it is the predominant prostanoid product excreted in the urine. Renal PGE2 have been established as being critically involved in regulating renal water transport, promoting urinary salt excretion and regulating blood pressure (18).

Substantial evidences suggest a natriuretic and diuretic role for PGE2 in the regulation of water balance. Previous studies in rabbit demonstrate that PGE2 directly inhibits Na+ absorption of collecting duct(CD) by increasing intracellular calcium *via* a pertussis toxin insensitive mechanism (19). Intrarenal and medullary infusion of PGE2 significantly increases urine sodium, consistent with its role as a natriuretic autacoid. However intravenous injection of PGE2 failed to produce this natriuretic effect, suggesting a local effect of PGE2 rather than a systemic effect (20). The concept that renal PGE2 antagonizes the actions of arginine vasopressin (AVP) in renal collecting ducts (CDs) had been described in a range of experiments. For example, in rabbit cortical collecting tubule, PGE2 inhibits AVP-induced cAMP activity and elevation of cytosolic calcium (Ca2+) *via* a G protein i(G_i_)-dependent mechanism (21). Besides, in rat renal inner medulla incubation with PGE2 reversed the AVP-induced aquaporin-2 (AQP2) membrane targeting without affecting AQP2 phosphorylation at ser-256 (22). In addition, Grazia and colleagues using sulprostone, a stable PGE2 analogue, stimulates Rho activity in inner medullary collecting duct(IMCD) cells resulting inhibited of AVP-induced AQP2 translocation, suggesting that activation of the G12/13/Rho pathway mediated diuretic action of PGE2 observed in the presence of AVP in a cAMP- and Ca2+-independent manner (23).

Paradoxically, PGE2 also plays a role in concentrating the urine. Increased PGE2 expression could be detected in urine and kidney in water deprivation mice. This increased expression of PGE2 is from up-regulated COX-2 and microsomal prostaglandin E synthase (mPGES)-1 (24). In addition to the antagonistic effects on AVP described above, *in vitro* and *in vivo* studies have shown that an increase in cellular water permeability can be detected as a result of elevated apical membrane abundance and phosphorylation of AQP2 induced by the PGE2 stimulus (25).

## PGE2 and Blood Pressure

It has been widely recognized that PGE2 is an important regulator of blood pressure, acting at different levels. However, as it exerts function through four different receptors, the mechanism is very complex. Systemic infusion of PGE2 results in a hypotensive effect, although in some circumstances it acts as a vasoconstrictor (26).

Blood pressure is downregulated in hypertensive rats and mice following inhibition of the prostaglandin transporter that mediates the inactivation of PGs (27). Results from PGE2 synthase knockout mice show that endogenous PGE2 is antihypertensive in salt-loading, angiotensin II, or aldosterone-induced hypertension models (23, 24, 28). In the renal microcirculation, PGE2 has been demonstrated to increase renal blood flow and glomerular filtration rate. Administration of PGE2 in wild-type mice resulted in an increase in afferent arteriolar diameter (29). In addition, PGE2 opposes Angiotensin II (Ang II) -induced accumulation of cytosolic calcium and ANG II-mediated constriction of renal pre-glomerular vascular smooth muscle cells (VSMCs) (30). The vasodilative effect of PGE2 on renal vessel is intended to maintain renal perfusion and urine volume under certain pathological states.

Other evidences ferret out vasoconstrictor effects of endogenous prostaglandins. PGE2 is thought to act centrally to elicit sympathetic excitation (31). Zhang et al. demonstrated that intracerebroventricular (ICV) administration of PGE2 elicited a sympathoexcitatory response, characterized by an increased blood pressure, and heart rate (31). In addition, Kazuo et al. found that PGE2 may play an important role in ICV administration of isoproterenol-induced elevations of plasma noradrenaline in the paraventricular nucleus (PVN) (32). In peripheral vascular, in absence of the vasodepressor EP receptor (EP2), PGE2 induces substantial hypertension (33).Many studies have indicated that PGE2 plays a role in the regulation of renal renin release (34, 35). The study of Anton Jan van Zonneveld et al. suggests that salt inducible MiR-132 regulates renin levels *via* COX-2/PGE2 *in vivo (* 36). Besides, it appears that PGE2 plays a dominant role in increasing tubular release of renin and the subsequent increase in angiotensin II in a COX-2-dependent manner, which is a support of blood pressure.

Together, these results suggest that PGE2 play a complex role in maintaining the body fluid balance and blood pressure homeostasis. These varied biological functions are regulated by four distinct G protein-coupled receptors. In order to figure out the inner mechanism of PGE2 in modulating the fluid and blood pressure homeostasis, it is necessary to find out the characteristics of each receptor. Thus, we summarize the distinct function of each individual EP receptor.

## PGE2 Receptors in Regulation of Fluid Balance and Blood Pressure

After synthesis, PGE2 leaves the cell and exerts its function *via* one of its four receptors in an autocrine or paracrine manner. Molecular identification of these receptors revealed that the prostanoid receptors are GPCRs. These GPCRs have seven transmembrane domains, an extracellular N terminus, and an intracellular carboxyl terminus and the transmembrane domains are connected by three intracellular and three extracellular loops. Prostaglandin E2 receptors (EPs) are termed as EP1, EP2, EP3, and EP4 receptor and are distinguished by amino acid identities, unique ligand binding profiles and different signal transduction properties (37, 38). PGE2 mediates diverse effects through EP receptors which mainly express in the kidney, vasculature and nervous system. In light of the side effect of NSAIDs, researchers committed to find out the particular role of each prostanoid receptor. Recent studies using specific antagonists and agonists of all four subtypes of EP receptors to disrupt various prostaglandin receptor genes alongside pharmacological studies has resulted in substantial progress in discerning the complex functions of these receptors in the vascular and renal segments.

## EP1 Receptor

The EP1 receptor mRNA is ubiquitously expressed and human EP1 receptor is comprised of 402 amino acids. EP1 receptor functions as constrictor in the smooth muscles of the trachea, gastrointestinal tract, bladder and uterus. Among the four subtypes of PGE2 receptors, EP1 receptor is unique because it is Gαq-coupled to mediate the mobilization of cytosolic Ca2+ and activation of protein kinase C (PKC) (39).

Studies in mouse, rabbit and human have shown that, mRNA expression of the renal EP1 receptor appears to be restricted to the collecting duct. However, in subsequent studies it was demonstrated that EP1 was also detected in glomerular mesangial cells, podocytes, and proximal tubule cells (40). With respect to a pathological role for EP1 signaling in the urine concentrating, pharmacological inhibition of the EP1 receptor completely blocked the PGE2-stimulated intracellular calcium increase and inhibition of Na+ absorption, suggesting a natriuretic effect (41). Na+-K+-ATPase in proximal tubule expels Na+ ions from the cell interior in exchange of K+ ions entering cell by ATP hydrolysis. In mouse renal tubular epithelial cell (MCT), Na+-K+-ATPase increased in PGE2 stimulation and the stimulatory effect was attenuated in EP1 or EP4 null cells with less Na+ excretion (40). A novel study shows that PGE2/EP1 attenuates AVP-H_2_O reabsorption in mouse IMCD and PGE2/EP1 inhibits sodium transport by both ENaC and pendrin-dependent pathways (42). An inhibitor of protein kinase C can partially reverse inhibition of water reabsorption and production of cAMP in cortical collecting ducts caused by PGE2. It implies that calcium signal is involved in the inhibition of vasopressin’s actions in these segments. In cultured IMCD cells and renal medulla, activation of EP1 receptor prevents expression of αENaC on both mRNA and protein level (43).

In regard to the role of EP1 receptor in blood pressure regulation, a variety of experiments show that it is a key player in hypertension and the end-organ damage, although its precise role is incompletely characterized. Disruption of EP1 receptor results in decreased mean arterial pressure, lessened aneurysm severity and the absence of anasarca, and a significant decrease in the incidence of mortality was observed in EP1+/+ but not EP1−/− mice, implicating that disruption of the EP1 receptor has a protective effect on end-organ damage (44).In some vascular beds, the pressor activity of EP1 receptor is implicated in the smooth muscle contractile response to PGE2. One prior study, using EP1-selective antagonist, AH6809, suggested that endogenous EP1 receptor, in part, contributes to the augmented pressure- and Ang II-induced arteriolar tone in mice with type 2 diabetes (45). Consistent with the vasoconstrictive property, studies utilizing mice with global targeted disruption of EP1 receptor exhibit a blunted pressor response to both acute and chronic Ang-II administration (46). Elevated blood pressure and prolonged vasoconstriction play a vital role in the inward remodeling of hypertension, the reduction in vascular stiffness and collagen deposition in patients with Ang-II infusion-induced hypertension in magnetic resonance angiography (MRA) resulted from the EP1 receptor blocker SC19220 further confirmed EP1 receptor in vascular injury in hypertension (47, 48). As for neurovascular, a recent study shows that constrictions of arterioles evoked by high concentrations of PGE2 were inhibited by SC51322, a specific antagonist of the PGE2 receptor subtype 1, suggesting that EP1 receptor has a vasoconstrictor effect on human cerebral parenchymal arterioles (49). In renal hemodynamics, EP1 null mice display elevated renin and aldosterone levels consistent with sustained activation of the renin-angiotensin- aldosterone system (RAAS) (50), although contradiction with the pressor function of EP1 receptor, one possibility is a self-regulation against systemic hypertension. Generalization, EP1 receptor causes constriction in peripheral and cerebral parenchymal arterioles and the vascular injury. Based on the aforementioned, targeting of EP1 receptors may provide novel therapeutic modalities for the treatment of hypertension.

## EP2 Receptor

The EP2 receptor shows broad tissue expression, including on kidney, smooth muscle, central nervous system (CNS), reproductive system and skeletal system (51). Although EP2 expression is minimal among the EP receptors, it can be efficiently induced by variety stimuli. The human EP2 receptor encoded a protein of 358 amino acids and is coupled to Gαs that activates adenylyl cyclase, resulting in the synthesis of cAMP from adenosine triphosphate (ATP) (39). Renal EP2 receptor mRNA is detected in glomeruli, vasa recta, outer and inner medulla of rat kidney (52)

In mice renal medulla, high-NaCl diet increases biosynthesis of COX2/mPGES1/PGE2. Intramedullary PGE2 infusion, but not intracortical infusion increase urinary sodium excretion. The EP2 agonist (Butaprost) can recur the natriuresis effect in renal medulla and this effect was abolished in EP2-deficient mice showing a natriuretic effect of EP2 receptor (20). As for the volume control of EP2 receptor. Both EP2 and vasopressin type 2 receptor(V2R) can increase cAMP by coupling to stimulatory G protein (Gs). Studies show an interaction between EP2 and AQP2. In Madin-Darby canine kidney (MDCK) cells, stimulating with butaprost causes AQP2 phosphorylation at ser-269 which help apical membrane retenion at the water channel with an increased intracellular cAMP as expected. This phenomenon will not be weaken even in rat treated with V2R antagonist means that it is independent of vasopressin and V2R (53). So, EP2 receptor was thought to alleviate polyuria of X-linked nephrogenic diabetes insipidus (NDI) for its pathogensis is mutations in the gene of vasopressin V2 receptor. Relevant animal experiments in rat model of nephrogenic diabetes insipidus (NDI), have conformed the potential treatment (25). These results combined suggest that stimulation of EP2 receptors may be another mechanism for increasing renal concentrating capacity.

Sodium excretion is a way to mitigate blood pressure. Although EP2-/- mice show lower systolic blood pressure compared to the EP2+/+ mice in standard (0.4% NaCl) diet. High-salt feeding (6% NaCl)failed to increases systolic blood pressure in EP2+/+ mice. However increased salt intake causes a rise in blood pressure of EP2-/- mice, showing that EP2 receptor may regulate sodium metabolism compromising salt-sensitive hypertension (33). Besides natriuresis, activation of EP2 receptor mediates the hypotensive function of PGE2 and the current consensus is that EP2 receptor acts as vasodilator. Previous studies demonstrate that targeted disruption of the EP2 receptor converts the dominant effect of PGE2 from a vasodepressor to a vasopressor (54, 55). The vasodilator role of prostaglandins is through increasing intracellular cAMP. Study by A. Mori et al. demonstrated that intravenous infusion of EP2 receptor agonist dilated retinal arterioles with increased capillary perfusion (56). As for the renal circulation, PGE2 or the EP2 receptor ligand butaprost causes an increase in afferent arteriolar diameter in WT mice but a decrease in vessel caliber in EP2-/- mice. Furthermore, in mice lacking the EP2 receptor, the vasoconstrictor response in afferent arteriolar was amplified by endothelin-1 (29). All of these show a vasodilatory role of the EP2 receptor and help to reduce blood pressure in organism.

## EP3 Receptor

Widely distributed in the smooth muscle of the gastrointestinal tract, the CNS, reproductive system, the kidney, the urinary bladder and vascular tissues, EP3 receptor is the most ubiquitous EP-receptor subtype among the four classes of EP receptors (51). The classic signaling pathway of the EP3 receptor is down-regulating cell membrane cAMP levels by coupling with inhibitory G protein(Gαi) (39). The uniqueness of this receptor lying in those multiple isoforms distinguished by amino acid composition in the C-terminal region. Three isoforms of mouse EP3 receptor (EP 3α, EP3β, EP3γ) and eight different splice mRNAs resulting in 5 isoforms of human EP3 receptor (EP3-Ia, EP3-Ib,EP3-Ic, EP3-II,EP3-III, EP3-IV, EP3-VI, and EP3-e) have been identified (37, 38, 57). It is cannot be overlooked that the splice variants of the EP3 receptor are linked to different signaling pathways including Gs(stimulation of intracellular cAMP formation),and Gq (stimulation of intracellular calcium), as well as coupling to G12/G13 (stimulation Rho kinase) (58). Because EP3 receptor-deficient (EP3 -/-)animals do not experience abnormalities in major organ systems and can survive in great numbers, studies on functions of EP3 receptor are mainly focused on EP3 -/- animals (59). And, due to overlapping tissue distribution of EP3 receptor isoforms and lack of specific agonists/antagonists, the diversity of each EP3-receptor isoform action has barely revealed.

In kidney, EP3 receptors are expressed at high levels in the renal medulla and cortical collecting duct, where PGE2 exerts its natriuretic and diuretic functions. Correspondingly, several animal models have been used to study the role of PGE2/EP3 in urine formation. For example, EP3−/− mice exhibit similar basal urine osmolality to wild-type (WT) mice. However, inhibition of PG synthesis by indomethacin was associated with a significant rise in urine osmolality in WT mice but not in EP3−/− mice, suggesting that PGE2/EP3 plays a role in modulating urine osmolality (59).Genetic deletion of the P2Y2 receptor significantly alleviates symptoms of lithium-induced polyuria, accompany with a marked decrease in EP3 receptor in the renal medulla and an increased in cellular cAMP levels. This result shows that EP3 receptor takes a place in P2Y2 induced diuresis by altering cellular cAMP levels (60). Since rat EP3-receptor isoforms are co-localized to renal distal tubules with vasopressin receptors, it is shown that rEP3a couples to Gi and rEP3b links to intracellular calcium inhibited vasopressin-induced antidiuretic action (59). Studies in mouse model of diabetes generated by streptozotocin show that cortical and medullary COX-2 protein and EP3 mRNA are upregulated during diabetes mellitus. The polydipsia is improved and urine osmolality is increased in Ep3−/− streptozotocin (STZ) mice. Compared to WT-STZ mice, there is also a significant increase in AQP1 in the medullary and AQP2 in the cortical and medullary in Ep3−/−STZ mice. The reverse in AVP-stimulated fluid reabsorption in WT-STZ mice led by Sulprostone is not seen in Ep3−/−STZ mice, further confirmed the negative role of EP3 in CD fluid reabsorption (61).

Not only the same effect as EP1 receptor in inhibiting fluid reabsorption, EP3 receptor knockout also resulted in lower blood pressure at baseline in mice. A study of Kazuo Ando et al. suggest that central isoproterenol-induced sympathetic cardiovascular responses such as hypertension and tachycardia weremediated *via* brain PGE2. Pretreatment with EP receptor antagonists reveled that this sympathoexcitatory response was depended on PGE2/EP3 receptor dependent manner (32). Acute infusion of the EP3 agonists resulted in a less increase in blood pressure in EP3−/− mice which is consistent with the vasopressor character of EP3 receptor. Different from EP1, EP3 receptor inhibits smooth muscle relaxation *via* mediating decreases in cAMP (62). Experimental studies in gene-disrupted mice show that EP3 receptor inactivation blunted the pressor response to AngII, however, EP3−/− mice exhibit intact hemodynamic responsiveness to other vasoactive agonists such as phenylephrine (PhE), sodium nitroprusside (SNP). Incubation of VSMCs with sulprostone potentiated the AngII-induced increase in intracellular Ca2+ levels, in contrast, the EP3 antagonist DG041 inhibited AngII-evoked Ca2+ signal. Besides, AngII treated mice showed increased EP3 receptor expression in cardiomyocytes. Systemic administration of EP3 antagonist L798,106 attenuated chronic AngII induced hypertension. All of these mentioned above suggest that the EP3 receptor may synergize AngII-induced vasoconstriction and targeting EP3 receptor is a new way to combat AngII-induced hypertension (63). Notably, in the peripheral vascular bed, including rat mesenteric artery, rat caudal artery, guinea pig aorta, human pulmonary artery and mouse kidney Vasculature EP3 receptors also display pressor effects (26). In renal microcirculation, blood flow and vascular resistance are increased in EP3-/- mice (64). In isolated proximal interlobular arteries, PGE2 elicited vasoconstriction response *via* EP3 receptor directly on smooth muscle cells but not endothelium and this phenomenon could not abolished by EP1 receptor antagonist (65). In mouse model of hypoxia-induced pulmonary hypertension(PAH), EP3 expression was strikingly elevated in pulmonary vessel wall and mouse EP3 receptor splice variants (EP3a and EP3b)expressed significantly increased in pulmonary arterial smooth muscle cells (PASMCs)compared with the normal condition. Further experiments unmasked a role of EP3 receptor in hypoxia-induced PAH model. To identified the G-protein coupled in, shRNAs designed to specifically knock down the expression of G12 or G13 suggest that EP3a/b variants mediate Rho-dependent TGF-β1 signaling in PASMCs in response to hypoxia *via* coupling to G12 (66). Pharmacological inhibition of EP3 receptor may be a supplemental treatment to PAH beside overexpression PGI2 or PGI2 analogs. Overall, selective inhibition of the EP3 receptor may provide benefits in blood pressure homeostasis.

## EP4 Receptor

In the kidney, a study in rats shows that the EP4 receptor was strongly expressed in the glomeruli, renin-secreting granular juxtaglomerular cells, distal convoluted tubules, cortical collecting ducts and vasa recta from the outer medulla (67). The EP4 receptor is a known regulator of urine concentration. In a viable mouse model of X-linked nephrogenic diabetes insipidus (XNDI) by deleting the V2 vasopressin receptor (V2R), mice administered with selective EP4 receptor agonist ONO-AE1-329 shows greatly improvements in the manifestations of XNDI, with significant reductions in urine volume and water intake while urine osmolality is massively increased (68). The same as V2R being the GPCR and coupled to Gs, EP4 may share the same pathway in antidiuresis. To test the hypothesis, studies by Emma and colleagues showed that an alternative EP4 receptor specific agonist (CAY10580) can partially mimic the effects of vasopression-V2R on AQP2 phosphorylation and membrane targeting in Madin-Darby canine kidney(MDCK) cells (25). *In vivo*, the Cre-loxP recombination system is adopted to generate EP4 renal tubule-specific knockout (Ksp-EP4−/−) mice and collecting duct-specific knockout (AQP2-EP4−/−) mice. AQP2 protein expression and phosphorylation at ser-256 were significantly reduced compared with controls under both hydrated and dehydrated conditions. There studies reveal that EP4 controls urinary concentration through regulation of AQP2 (24)..

To further investigate the detailed mechanism responsible for EP4 regulation of AQP2. Wang and colleagues proposed a possibility of (Pro) renin receptor (PRR) binding renin and prorenin as the regulator between EP4 and AQP2. COX-2 and EP4 are important regulator of renal medullary PRR expression. PRR increases urine osmolality and AQP2 expression in water deprivation rat. The antidiuretic action was abolished by EP4 antagonist ONO-AE3-208, so as renal PRR expression. In their study, they also figure out EP4 receptor signal through cAMP/PKA regulates PRR expression and the following AQP2 transcription and translocation (69). However, study by Rolf at el. shows that EP4 may be involved in the pathogenesis of hyperprostaglandin E syndrome/antenatal Bartter syndrome (HPS/aBS), and gene target disruption of EP4-/- on mixed background mice suppresses furosemide induced diuresis and electrolyte excretion, suggesting a natriuretic role of EP4 (70).

Global EP4 gene deletion mice are complicated by patent ductus arteriosus which is perinatally lethal, indicating a critical role of EP4 receptor in physiological vascular function (71). Vascular smooth muscle cell-specific EP4 gene knockout (VSMC-EP4-/-) mice exhibited higher blood pressure level in chronic AngII infusion compared with the WT mice showing a role of EP4 receptor in blood pressure homeostasis (72). Previous studies have revealed that EP4 receptor belongs to relaxant receptor, which mediate increases in cAMP and induce vascular smooth muscle relaxation. In vascular endothelial cells (ECs), EP4 receptor mediated PGE2-elicited acute vasodilator response is dependent on endothelium derived NO production by coupling with endothelial nitric oxide synthase (eNOS) (73, 74). The EP4 agonist PGE1-OH and CAY10580 markedly reduced BP levels in Dahl salt-sensitive hypertensive rats (75). In renal vascular system, EP4 receptor play a paradoxical role in activating renin-angiotensin-aldosterone system. RAAS plays a key physiological role in the regulation of blood pressure, electrolyte homeostasis and kidney development. The rate-limiting step of the downstream activity of the system depends on the level of renin. Compared with EP4+/+ mice, renin activity in salt deprivation mice was impaired in EP4-/- mouse. Renin mRNA expression was decreased in wild-type mouse treated with the selective EP4 receptor antagonist compared with EP4+/+ mice on a low-salt diet. This experiment illustrated that EP4 receptor directly enhance renin secretion in response to salt deprivation *in vivo* (76). However, the mean arterial pressure in anesthetized mice was not significantly different. These findings are not readily explained. Perhaps due to compensatory increases in activity of the renin-angiotensin system. Compared to other EP receptors, experiments by Carie and associates suggested the Gs-coupled EP4 receptor as the most important receptor for stimulation of renin (77). Taken together, EP4 receptor acts on both endothelial cells (ECs) and vascular smooth muscle cells (VSMCs) as a vasodilator. Vascular and renal EP4 receptors sustain the fluid balance through respective local actions. EP4 receptor analogs may become attractive hypertensive treatment.

## Applications of EP Antagonists

Given the side effect of NSAIDs (78), and the convolution of targeting the inducible mPGES-1 to inhibit the PGE2 synthesis from COX-2 based on the interspecies differences in the sequence and structure, EPs antagonists arouse extensive development. Here, we summarize the EPs agonists/antagonists in the regulation of fluid balance and blood pressure mentioned above (**Table 1**). However, there is no human clinical trial on EPs antagonists in fluid balance and blood pressure homeostasis. Studies using other disease models revealed the possible therapeutic applications of those EPs antagonists, and some of them are currently available in clinical trials (**Table 2**, https://clinicaltrials.gov/). It is well known that the selectivity of prostanoids for their respective receptors is not absolute, so as the EPs antagonists. Due to the sequence homology of EPs antagonists, some compounds play a dual antagonistic role. High potency, selectivity and oral bioavailability EPs agonists/antagonists should be taken into consideration.

**Table 1:** Roles of EP receptors agonists/antagonists in the regulation of fluid balance and blood pressure.

Reagent	Function in fluid metabolism	Function in Blood Pressure	Reference
Agonists			
Butaprost (EP2)	Natriuresis Antidiuresis	Increase afferent arteriolar flow	(25, 53)
Sulprostone (EP3>EP1)	Diuresis	Increase blood pressure	(64)
ONO-AE1-329 (EP4)	Natriuresis, Antidiuresis	No data	(68)
CAY10580 (EP4)	Antidiuresis	Reduce blood pressure	(25)
PGE1-OH (EP4)	No data	Reduce blood pressure	(75)
Antagonists			
SC19220 (EP1)	No data	Reduce blood pressure Reduce vascular injury	(47)
AH6809 (EP1)	No data	Reduce blood pressure	(38)
DG-041(EP3)	No data	Reduce blood pressure	(38)
L798,106(EP3)	No data	Reduce blood pressure	(63)
ONO-AE3-208(EP4)	Diuresis	No data	(59)

**Table 2:** Clinical trials of EP receptors agonists/antagonists.

Reagent	Sponsor	Indication	Phase	NCT number
Agonists				
Sulprostone(EP3)	Assistance Publique - Hôpitaux de Paris	Postpartum Hemorrhage	–	NCT02118038
Sulprostone(EP3)	Atrium Medical Center	Abortion	–	NCT00206193
ONO-AE1-734 (EP4)	Kyoto University, Graduate School of Medicine	Ulcerative Colitis	Phase2	NCT00296556
Antagonists				
CJ-023,423(EP4)	Pfizer	Osteoarthritis	Phase1	NCT00392080
BGC20-1531(EP4)	BTG International Inc.	Migraine	Phase2	NCT00888680
ONO-8539 (EP1)	Hyogo College of Medicine	Gastroesophageal Reflux Disease		UMIN000015753

## Conclusion

In summary, PGE2 as a lipid mediator regulates fluid and blood pressure homeostasis dynamically. Intrarenal infusion of PGE2 presents a natriuretic effect while has a function of concentrating the urine under physiological conditions. PGE2 shows both positive and negative effect on blood pressure regulation. Modulation of the divergent roles of PGE2 is enabled by multiple receptors with diverse signaling abilities(**Table 3**; **Figure 1**). Among the four EP receptor, EP1 receptor accounts for the major natriuretic effect of PGE2.EP3 receptor mRNA is expressed highly in the medullary and the collecting duct and is involved in the pathogenesis of various water deletion diseases, such as NDI and diabetes mellitus. Both EP1 and EP3 receptors inhibit renal fluid reabsorption. EP2 and EP4 receptors share the same Gs proteins and work in correspondence with AQP2, contributing to fluid reabsorption. In mitigating blood pressure, EP1 receptor couples to increased intracellular Ca^2+^ implicating in the contractile response to PGE2. EP3 receptor activation decreases intracellular cAMP generation *via* Gi protein and inhibits vasodilatation. In contrast, EP2 and EP4 receptors coupled to Gs-proteins mediate increases in intracellular cAMP and induce vasodilatation. In summary, PGE2 upregulates blood pressure through EP1 and EP3 receptors, while it downregulates blood pressure through EP2 and EP4 receptors. PGE2 and EP receptors may mediate contradictory actions in regional of kidney or blood vessel to control systemic hemodynamics. An in-depth understanding of these specific EP receptors may provide unique opportunity for development of promising targets for modulating renal salt and water excretion as well as systemic BP.

**Table 3:** Roles of EP receptors in the regulation of fluid balance and blood pressure.

Type	G Protein	Second Messenger	Function in Fluid Metabolism	Function in Blood Pressure
EP1	Gαq	Ca2+↑, PKC↑	Natriuresis and Inhibit Fluid Reabsorption	Vasoconstriction; *increase blood pressure*
EP2	Gαs	cAMP↑	Natriuresis and Antidiuresis	Vasodilatation, Increase Renal Blood Flow; *decrease blood pressure*
EP3	Gαi Gαq Gαs	cAMP↓, Ca2+↑, cAMP↑	Natriuresis and Inhibit Fluid Reabsorption	Inhibit Vasodilatation, Synergize AngII-induced Vasoconstriction; *increase blood pressure*
EP4	Gαs	cAMP↑	Natriuresis and Antidiuresis	Vasodilatation; *decrease blood pressure*

"↑" activated; "↓" inhibited.

## Author Contributions

LW and YW are the co-first authors. SH and JY are co-corresponding authors. LW, YW, JY and SH wrote this paper. SH, JY and ZJ designed and revised it. All authors contributed to the article and approved the submitted version.

## Conflict of Interest

The authors declare that the research was conducted in the absence of any commercial or financial relationships that could be construed as a potential conflict of interest.

## Publisher’s Note

All claims expressed in this article are solely those of the authors and do not necessarily represent those of their affiliated organizations, or those of the publisher, the editors and the reviewers. Any product that may be evaluated in this article, or claim that may be made by its manufacturer, is not guaranteed or endorsed by the publisher.
